# The 100 Most Cited Papers Concerning the Insular Cortex of the Brain: A Bibliometric Analysis

**DOI:** 10.3389/fnhum.2018.00337

**Published:** 2018-08-27

**Authors:** Andy W. K. Yeung

**Affiliations:** Oral and Maxillofacial Radiology, Applied Oral Sciences, Faculty of Dentistry, The University of Hong Kong, Hong Kong, Hong Kong

**Keywords:** bibliometric, brain, insular cortex, insula of Reil, neurosciences, publications

## Abstract

**Background:** The insula is one of the most researched brain regions with many highly cited papers. However, unlike the literature of other fields, there is currently no study that has identified the 100 most cited papers within the literature of the insula. The aim of the current study was to fill in the knowledge gap by determining which publications concerning the insula have been cited most often, who contributed to them, and what topics they were dealing with.

**Methods:** The Web of Science online database was searched to identify the 100 most cited publications mentioning the insular cortex in their titles, abstracts or keywords. To systematically exclude irrelevant publications, the search strategy was finalized as: TS = (insula OR insular OR “island of Reil”) NOT TS = (“insular biogeography” OR “insular mammal^*^” OR “^*^insular lymphatic^*^”) NOT WC = (“Geochemistry and Geophysics” OR “Ecology”). The identified publications were sorted in descending order of citation count. The 100 most cited publications concerning the insula of the brain were identified and their bibliometric data was extracted and assessed. The VOSviewer software was used with default parameters to generate a bubble map that analyzes and visualizes the words/phrases used in the titles and abstracts of the publications.

**Results:** There were 67 articles on experiments/lab studies and 33 meta-analyses/reviews but no opinion or methods paper. They had an average of 943.4 citations (or 62.9 citations per year), 93.5 references and 13.4 pages. There were 35 papers published in open access. USA was the major contributing country. The most top-ranked publications were concerning emotion, salience and pain.

**Conclusion:** Two-thirds of the publications concerned the normal brain function/mechanism (*n* = 67), whereas 20 publications concerned disease/therapeutic intervention and another 13 concerned normal anatomy. For the 67 original articles, 57 used human subjects whereas 10 used animal models. MRI was the commonest modality (*n* = 37), followed by PET (*n* = 16). Nine articles investigated by histology, two by multiple modalities and three by other modality.

## Introduction

The word “insula” was firstly coined by Professor Reil and the insula is also known as “Island of Reil” (Reil, 2002; Binder et al., 2007). It is a fascinating structure of the cerebral cortex that has multiple functions and clinical relevance. For instance, it is involved in sensory processing (Naghavi et al., 2007; Yeung et al., 2016, 2018a), motor control (Ackermann and Riecker, 2004), interoceptive awareness (Naqvi and Bechara, 2010), autonomic control (Yasui et al., 1991), and social emotions (Lamm and Singer, 2010). It is often investigated in clinical topics such as addiction (Naqvi and Bechara, 2009), anorexia nervosa (Wagner et al., 2008), and stroke (Cereda et al., 2002). In short, the insula is involved in many researches, ranging from neurology, neuropathology, psychiatry, and psychology.

The evaluation of the academic impact of the published literature in terms of citation count has been gaining attention. It not only allows the evaluation of research performance, but also helps investigators refine their research blueprint based on the research trends and overall landscape of the research field (Brinjikji et al., 2013; Yeung et al., 2017a). There are already numerous reports that identified the 100 most cited articles, also known as citation classics, in various fields such as imaging (Brinjikji et al., 2013), radiology (Yoon et al., 2013; Pagni et al., 2014), neuroimaging (Kim et al., 2016), neurosurgery (Ponce and Lozano, 2010), and neurosciences (Yeung et al., 2017a). Analyses based on citation counts are primarily supported by an earlier notion that citation counts could be correlated to peer judgments (Bayer and Folger, 1966; Garfield, 1970), which are in turn commonly accepted as a way to rank scientific performance (Garfield, 1979). Citation counts are not without problems. For instance, MacRoberts and MacRoberts (1989) have pointed out that potential problems of citation analysis included inability to assess formal and informal influences not expressed as journal citations, biased citation behavior, self-citation, different meaning of each citation, variations in citation rates due to various background parameters of a publication, and technical shortcomings of citation databases. Nonetheless, citation analysis and journal impact factors are used in many circumstances, such as determining departmental funding in the universities, assessing research grant proposals, and deciding promotions and appointments of academic staff (Adam, 2002). Therefore, results reported in bibliometric studies enabled researchers to quickly identify the most cited publications of the respective field so that they might further the research works or develop new research directions based on these cornerstones (Khan et al., 2017). However, such bibliometric evaluation has not been applied to the research of the insula, a versatile multifunctional brain region and a research hot topic with highly cited papers (Yeung et al., 2017c; Yeung, 2018). Therefore, the aim of the current study was to fill in the knowledge gap by determining which publications concerning the insula have been cited most often, who contributed to them, and what topics they were dealing with. In particular, the current study tried to analyze what factors were related to the citation counts, e.g., normalized citation count, reference count, usage count, years since publication, number of authors, page count, whether the publications were in open access, the publication type, study topic, the study model, and the modality used.

## Materials and methods

### Search method and strategy

The search was performed on 18 July 2018 using the Web of Science (WoS) online database hosted by Clarivate Analytics. Based on a pilot search, the author found that some of the highly cited publications mentioning the word “insula” or its derivatives are not related to the insular cortex of the brain, such as those concerning insular biogeography (investigation of animals and plants in isolated natural environments) and those concerning peri-insular lymphatics of the pancreas. To systematically exclude these irrelevant publications instead of having a subjective manual exclusion, the search strategy was finalized as: TS = (insula OR insular OR “island of Reil”) NOT TS = (“insular biogeography” OR “insular mammal^*^” OR “^*^insular lymphatic^*^”) NOT WC = (“Geochemistry and Geophysics” OR “Ecology”). It identified publications mentioning the insula in their titles, abstracts, author keywords or KeyWord Plus (keywords indexed by WoS). Keywords serve the function of providing rapid access to scientific works (Soos et al., 2013) and are useful for bibliometric analyses (Stock and Stock, 2013; Zhang et al., 2016; Vargas-Quesada et al., 2017), and thus were considered together with abstracts and titles. No other restriction was placed on the search, such as publication year. The identified publications were sorted in descending order of citation count. The 100 most cited publications concerning the insula of the brain were identified and their bibliometric data was extracted and assessed, including the author list, affiliation, country of affiliation, journal, language, WoS category of the publication, publication year, number of references, number of citations, number of pages of the manuscript, and status of open access, the publication type (experiment/lab studies, methods, meta-analysis/review/opinion), and the study topic (normal brain function/mechanism, disease/therapeutic intervention, normal anatomy). For experiment/lab studies, the study model (human, animal, computational, multiple) and modality [magnetic resonance imaging (MRI), positron emission tomography (PET), multiple, histology, others] were also assessed.

### Network visualization

VOSviewer, software designed to visualize bibliometric data (Van Eck and Waltman, 2009), was used to generate a bubble map that analyzes and visualizes the words/phrases used in the titles and abstracts of the 100 publications. All words/phrases that appeared in two or more of the 100 publications were included. Binary counting was used, implying that multiple appearances in a single publication counted as one. Default visualization parameters were chosen. Two bubbles were in closer proximity if they co-appeared in a larger number of publications. The number of publications in which the word/phrase appeared determined the bubble size. The bubble color was determined by the averaged citations per publication containing the word/phrase. This method has been successfully used to analyze the titles and abstracts of publications from neuroimaging, neurosciences, food sciences and pharmacology (Yeung et al., 2017b,c, 2018b,c; Yeung, 2018).

### Statistical analysis

Exploratory analysis was performed to evaluate if the citation count had significant associations with the numbers of normalized citation count (citations per year after publication), years since publication, references, pages, authors, as well as the usage count since 2013 (i.e., the number of times the full text of a publication was accessed or saved through WoS), status of open access, publication type, study topic, study model, and modality. Pearson's correlation tests were conducted to assess the former six variables, whereas two-sample *t* test was conducted to assess the latter five variables. For the last four variables, either only two groups had values or the groupings were binarized (e.g., study topic binarized into findings from healthy brains/patients and from patients with diseases/disorders; and modality binarized into MRI and non-MRI). The statistical analysis was performed with SPSS 25.0 (IBM, New York, USA). Test results with *p* < 0.05 were considered statistically significant.

## Results

### The overview

The publication years of top 100 most cited publications of the insula were happened to be from 1982 to 2011. The list was presented in Table 1. There were 67 articles on experiments/lab studies and 33 meta-analyses/reviews but no opinion or methods paper. They have received a total of 94,340 citations from 61,624 citing articles, equaling an average of 943.4 citations (*SD* = 506.2, range = 544–2924). When the citation counts were normalized by the number of years since publication, the average value became 62.9 (*SD* = 46.3, range = 17.9–268.7). The usage count since 2013 through WoS had an average of 140 times (SD = 143, range = 6–680). On average they had a whopping number of 93.5 references (SD = 73.4, range = 19–387). The average length of the manuscript was 13.4 pages (SD = 8.3, range = 3–47). There were 35 papers published in open access (gold, bronze or green open access). All of them were written in English. Two-thirds of the publications concerned the normal brain function/mechanism (*n* = 67), whereas 20 publications concerned disease/therapeutic intervention and another 13 concerned normal anatomy.

**Table 1:** The top 100 citation classics concerning the insula of the brain.

**Rank**	**Paper**	**No. of references**	**Citation count**	**Citations per year**
1	Good, C. D., Johnsrude, I., Ashburner, J., Henson, R. N., Friston, K. J., and Frackowiak, R. S. (2001). A voxel-based morphometric study of aging in 465 normal adult human brains. *NeuroImage* 14, 21–36.	66	2,924	172.0
2	Seeley, W. W., Menon, V., Schatzberg, A. F., Keller, J., Glover, G. H., Kenna, H., …and Greicius, M. D. (2007). Dissociable intrinsic connectivity networks for salience processing and executive control. *Journal of Neuroscience, 27*(9), 2349-2356.	64	2,523	229.4
3	Craig, A. D., and Craig, A. D. (2009). How do you feel–now? The anterior insula and human awareness. *Nature Reviews Neuroscience, 10*(1), 59-70.	128	2,418	268.7
4	Cabeza, R., and Nyberg, L. (2000). Imaging cognition II: An empirical review of 275 PET and fMRI studies. *Journal of Cognitive Neuroscience, 12*(1), 1-47.	386	2,320	128.9
5	Craig, A. D. (2002). How do you feel? Interoception: the sense of the physiological condition of the body. *Nature Reviews Neuroscience, 3*(8), 655-666.	159	2,313	144.6
6	Devinsky, O., Morrell, M. J., and Vogt, B. A. (1995). Contributions of anterior cingulate cortex to behavior. *Brain, 118*(1), 279-306.	246	2,222	96.6
7	Phan, K. L., Wager, T., Taylor, S. F., and Liberzon, I. (2002). Functional neuroanatomy of emotion: a meta-analysis of emotion activation studies in PET and fMRI. *NeuroImage, 16*(2), 331-348.	131	1,944	121.5
8	Koob, G. F., and Volkow, N. D. (2010). Neurocircuitry of addiction. *Neuropsychopharmacology, 35*(1), 217-238.	239	1,896	237.0
9	Singer, T., Seymour, B., O'doherty, J., Kaube, H., Dolan, R. J., and Frith, C. D. (2004). Empathy for pain involves the affective but not sensory components of pain. *Science, 303*(5661), 1157-1162.	38	1,884	134.6
10	Bechara, A., Damasio, H., and Damasio, A. R. (2000). Emotion, decision making and the orbitofrontal cortex. *Cerebral Cortex, 10*(3), 295-307.	62	1,695	94.2
11	Critchley, H. D., Wiens, S., Rotshtein, P., Öhman, A., and Dolan, R. J. (2004). Neural systems supporting interoceptive awareness. *Nature Neuroscience, 7*(2), 189-195.	47	1,559	111.4
12	Mayberg, H. S., Liotti, M., Brannan, S. K., McGinnis, S., Mahurin, R. K., Jerabek, P. A., …and Fox, P. T. (1999). Reciprocal limbic-cortical function and negative mood: converging PET findings in depression and normal sadness. *American Journal of Psychiatry, 156*(5), 675-682.	59	1,521	80.1
13	Menon, V., and Uddin, L. Q. (2010). Saliency, switching, attention and control: a network model of insula function. *Brain Structure and Function, 214*(5-6), 655-667.	93	1,519	189.9
14	Sanfey, A. G., Rilling, J. K., Aronson, J. A., Nystrom, L. E., and Cohen, J. D. (2003). The neural basis of economic decision-making in the ultimatum game. *Science, 300*(5626), 1755-1758.	28	1,471	98.1
15	Apkarian, A. V., Bushnell, M. C., Treede, R. D., and Zubieta, J. K. (2005). Human brain mechanisms of pain perception and regulation in health and disease. *European Journal of Pain, 9*(4), 463-484.	239	1,412	108.6
16	Etkin, A., and Wager, T. D. (2007). Functional neuroimaging of anxiety: a meta-analysis of emotional processing in PTSD, social anxiety disorder, and specific phobia. *American Journal of Psychiatry, 164*(10), 1476-1488.	119	1,403	127.5
17	Peyron, R., Laurent, B., and Garcia-Larrea, L. (2000). Functional imaging of brain responses to pain. A review and meta-analysis (2000). *Neurophysiologie Clinique/Clinical Neurophysiology, 30*(5), 263-288.	168	1,292	71.8
18	Cardinal, R. N., Parkinson, J. A., Hall, J., & Everitt, B. J. (2002). Emotion and motivation: the role of the amygdala, ventral striatum, and prefrontal cortex. *Neuroscience & Biobehavioral Reviews, 26*(3), 321-352.	374	1,257	78.6
19	Phillips, M. L., Drevets, W. C., Rauch, S. L., & Lane, R. (2003). Neurobiology of emotion perception I: The neural basis of normal emotion perception. *Biological Psychiatry, 54*(5), 504-514.	144	1,245	83.0
20	Corbetta, M., Miezin, F. M., Dobmeyer, S., Shulman, G. L., & Petersen, S. E. (1991). Selective and divided attention during visual discriminations of shape, color, and speed: functional anatomy by positron emission tomography. *Journal of Neuroscience, 11*(8), 2383-2402.	133	1,204	44.6
21	Raichle, M. E., Fiez, J. A., Videen, T. O., MacLeod, A. M. K., Pardo, J. V., Fox, P. T., & Petersen, S. E. (1994). Practice-related changes in human brain functional anatomy during nonmotor learning. *Cerebral Cortex, 4*(1), 8-26.	90	1,122	46.8
22	Wicker, B., Keysers, C., Plailly, J., Royet, J. P., Gallese, V., & Rizzolatti, G. (2003). Both of us disgusted in My insula: the common neural basis of seeing and feeling disgust. *Neuron, 40*(3), 655-664.	64	1,107	73.8
23	Augustine, J. R. (1996). Circuitry and functional aspects of the insular lobe in primates including humans. *Brain Research Reviews, 22*(3), 229-244.	81	1,091	49.6
24	Dosenbach, N. U., Fair, D. A., Miezin, F. M., Cohen, A. L., Wenger, K. K., Dosenbach, R. A., …& Schlaggar, B. L. (2007). Distinct brain networks for adaptive and stable task control in humans. *Proceedings of the National Academy of Sciences of the United States of America, 104*(26), 11073-11078.	55	1,086	98.7
25	Carr, L., Iacoboni, M., Dubeau, M. C., Mazziotta, J. C., & Lenzi, G. L. (2003). Neural mechanisms of empathy in humans: a relay from neural systems for imitation to limbic areas. *Proceedings of the National Academy of Sciences of the United States of America, 100*(9), 5497-5502.	60	1,059	70.6
26	Phillips, M. L., Young, A. W., Senior, C., Brammer, M., Andrew, C., Calder, A. J., …& Gray, J. A. (1997). A specific neural substrate for perceiving facial expressions of disgust. *Nature, 389*(6650), 495-498.	30	1,050	50.0
27	Gallese, V., Keysers, C., & Rizzolatti, G. (2004). A unifying view of the basis of social cognition. *Trends in Cognitive Sciences, 8*(9), 396-403.	68	1,046	74.7
28	Phillips, M. L., Drevets, W. C., Rauch, S. L., & Lane, R. (2003). Neurobiology of emotion perception II: implications for major psychiatric disorders. *Biological Psychiatry, 54*(5), 515-528.	147	1,032	68.8
29	Craig, A. D. (2003). Interoception: the sense of the physiological condition of the body. *Current Opinion in Neurobiology, 13*(4), 500-505.	55	1,028	68.5
30	Wager, T. D., Rilling, J. K., Smith, E. E., Sokolik, A., Casey, K. L., Davidson, R. J., …& Cohen, J. D. (2004). Placebo-induced changes in FMRI in the anticipation and experience of pain. *Science, 303*(5661), 1162-1167.	37	1,026	73.3
31	Davidson, R. J., & Irwin, W. (1999). The functional neuroanatomy of emotion and affective style. *Trends in Cognitive Sciences, 3*(1), 11-21.	101	1,001	52.7
32	Adolphs, R. (2002). Neural systems for recognizing emotion. *Current Opinion in Neurobiology, 12*(2), 169-177.	99	994	62.1
33	Sridharan, D., Levitin, D. J., & Menon, V. (2008). A critical role for the right fronto-insular cortex in switching between central-executive and default-mode networks. *Proceedings of the National Academy of Sciences of the United States of America, 105*(34), 12569-12574.	53	982	98.2
34	Arnold, S. E., Hyman, B. T., Flory, J., Damasio, A. R., & Van Hoesen, G. W. (1991). The topographical and neuroanatomical distribution of neurofibrillary tangles and neuritic plaques in the cerebral cortex of patients with Alzheimer's disease. *Cerebral Cortex, 1*(1), 103-116.	74	923	34.2
35	Garavan, H., Ross, T. J., & Stein, E. A. (1999). Right hemispheric dominance of inhibitory control: an event-related functional MRI study. *Proceedings of the National Academy of Sciences of the United States of America, 96*(14), 8301-8306.	49	911	47.9
36	Morris, J. S., Friston, K. J., Büchel, C., Frith, C. D., Young, A. W., Calder, A. J., & Dolan, R. J. (1998). A neuromodulatory role for the human amygdala in processing emotional facial expressions. *Brain, 121*(1), 47-57.	76	852	42.6
37	Dosenbach, N. U., Visscher, K. M., Palmer, E. D., Miezin, F. M., Wenger, K. K., Kang, H. C., …& Petersen, S. E. (2006). A core system for the implementation of task sets. *Neuron, 50*(5), 799-812.	81	849	70.8
38	Price, C. J. (2000). The anatomy of language: contributions from functional neuroimaging. *Journal of Anatomy, 197*(3), 335-359.	125	849	47.2
39	Breiter, H. C., Gollub, R. L., Weisskoff, R. M., Kennedy, D. N., Makris, N., Berke, J. D., …& Mathew, R. T. (1997). Acute effects of cocaine on human brain activity and emotion. *Neuron, 19*(3), 591-611.	113	844	40.2
40	Sweatt, J. D. (2001). The neuronal MAP kinase cascade: a biochemical signal integration system subserving synaptic plasticity and memory. *Journal of Neurochemistry, 76*(1), 1-10.	65	815	47.9
41	Chollet, F., DiPiero, V., Wise, R. J. S., Brooks, D. J., Dolan, R. J., & Frackowiak, R. S. J. (1991). The functional anatomy of motor recovery after stroke in humans: a study with positron emission tomography. *Annals of Neurology, 29*(1), 63-71.	37	804	29.8
42	Singer, T., Seymour, B., O'doherty, J. P., Stephan, K. E., Dolan, R. J., & Frith, C. D. (2006). Empathic neural responses are modulated by the perceived fairness of others. *Nature, 439*(7075), 466.	29	784	65.3
43	Brog, J. S., Salyapongse, A., Deutch, A. Y., & Zahm, D. S. (1993). The patterns of afferent innervation of the core and shell in the “accumbens” part of the rat ventral striatum: immunohistochemical detection of retrogradely transported fluoro-gold. *Journal of Comparative Neurology, 338*(2), 255-278.	122	781	31.2
44	Gorno-Tempini, M. L., Dronkers, N. F., Rankin, K. P., Ogar, J. M., Phengrasamy, L., Rosen, H. J., …& Miller, B. L. (2004). Cognition and anatomy in three variants of primary progressive aphasia. *Annals of Neurology, 55*(3), 335-346.	74	756	54.0
45	Suzuki, W. L., & Amaral, D. G. (1994). Perirhinal and parahippocampal cortices of the macaque monkey: cortical afferents. *Journal of Comparative Neurology, 350*(4), 497-533.	108	747	31.1
46	Oppenheimer, S. M., Gelb, A., Girvin, J. P., & Hachinski, V. C. (1992). Cardiovascular effects of human insular cortex stimulation. *Neurology, 42*(9), 1727-1727.	25	743	28.6
47	Ploghaus, A., Tracey, I., Gati, J. S., Clare, S., Menon, R. S., Matthews, P. M., & Rawlins, J. N. P. (1999). Dissociating pain from its anticipation in the human brain. *Science, 284*(5422), 1979-1981.	25	741	39.0
48	Carmichael, S. T., & Price, J. L. (1995). Limbic connections of the orbital and medial prefrontal cortex in macaque monkeys. *Journal of Comparative Neurology, 363*(4), 615-641.	114	739	32.1
49	Bandler, R., & Shipley, M. T. (1994). Columnar organization in the midbrain periaqueductal gray: modules for emotional expression?. *Trends in Neurosciences, 17*(9), 379-389.	71	735	30.6
50	Vertes, R. P. (2004). Differential projections of the infralimbic and prelimbic cortex in the rat. *Synapse, 51*(1), 32-58.	192	725	51.8
51	Date, Y., Ueta, Y., Yamashita, H., Yamaguchi, H., Matsukura, S., Kangawa, K., …& Nakazato, M. (1999). Orexins, orexigenic hypothalamic peptides, interact with autonomic, neuroendocrine and neuroregulatory systems. *Proceedings of the National Academy of Sciences of the United States of America, 96*(2), 748-753.	19	719	37.8
52	Dronkers, N. F. (1996). A new brain region for coordinating speech articulation. *Nature, 384*(6605), 159-161.	29	717	32.6
53	Jahanshahi, M., Jenkins, I. H., Brown, R. G., Marsden, C. D., Passingham, R. E., & Brooks, D. J. (1995). Self-initiated versus externally triggered movements: I. An investigation using measurement of regional cerebral blood flow with PET and movement-related potentials in normal and Parkinson's disease subjects. *Brain, 118*(4), 913-933.	82	710	30.9
54	Braun, A. R., Balkin, T. J., Wesenten, N. J., Carson, R. E., Varga, M., Baldwin, P., …& Herscovitch, P. (1997). Regional cerebral blood flow throughout the sleep-wake cycle. An H2(15)O PET study. *Brain, 120*(7), 1173-1197.	117	709	33.8
55	Goldin, P. R., McRae, K., Ramel, W., & Gross, J. J. (2008). The neural bases of emotion regulation: reappraisal and suppression of negative emotion. *Biological Psychiatry, 63*(6), 577-586.	51	703	70.3
56	Bartels, A., & Zeki, S. (2004). The neural correlates of maternal and romantic love. *NeuroImage, 21*(3), 1155-1166.	92	701	50.1
57	Mayberg, H. S., Brannan, S. K., Tekell, J. L., Silva, J. A., Mahurin, R. K., McGinnis, S., & Jerabek, P. A. (2000). Regional metabolic effects of fluoxetine in major depression: serial changes and relationship to clinical response. *Biological Psychiatry, 48*(8), 830-843.	121	696	38.7
58	Balleine, B. W., & Dickinson, A. (1998). Goal-directed instrumental action: contingency and incentive learning and their cortical substrates. *Neuropharmacology, 37*(4-5), 407-419.	50	696	34.8
59	Resnick, S. M., Pham, D. L., Kraut, M. A., Zonderman, A. B., & Davatzikos, C. (2003). Longitudinal magnetic resonance imaging studies of older adults: a shrinking brain. *Journal of Neuroscience, 23*(8), 3295-3301.	40	685	45.7
60	Menon, V., Adleman, N. E., White, C. D., Glover, G. H., & Reiss, A. L. (2001). Error-related brain activation during a Go/NoGo response inhibition task. *Human Brain Mapping, 12*(3), 131-143.	62	680	40.0
61	Critchley, H. D. (2005). Neural mechanisms of autonomic, affective, and cognitive integration. *Journal of Comparative Neurology, 493*(1), 154-166.	96	670	51.5
62	Shin, L. M., & Liberzon, I. (2010). The neurocircuitry of fear, stress, and anxiety disorders. *Neuropsychopharmacology, 35*(1), 169-191.	387	667	83.4
63	Small, D. M., Zatorre, R. J., Dagher, A., Evans, A. C., & Jones-Gotman, M. (2001). Changes in brain activity related to eating chocolate: from pleasure to aversion. *Brain, 124*(9), 1720-1733.	89	665	39.1
64	Weiller, C., Chollet, F., Friston, K. J., Wise, R. J., & Frackowiak, R. S. (1992). Functional reorganization of the brain in recovery from striatocapsular infarction in man. *Annals of Neurology, 31*(5), 463-472.	37	663	25.5
65	Treede, R. D., Kenshalo, D. R., Gracely, R. H., & Jones, A. K. (1999). The cortical representation of pain. *Pain, 79*(2-3), 105-111.	56	658	34.6
66	Fusar-Poli, P., Placentino, A., Carletti, F., Landi, P., Allen, P., Surguladze, S., …& Perez, J. (2009). Functional atlas of emotional faces processing: a voxel-based meta-analysis of 105 functional magnetic resonance imaging studies. *Journal of Psychiatry & Neuroscience, 34*(6), 418-432.	177	649	72.1
67	Mesulam, M. M., & Mufson, E. J. (1982). Insula of the old world monkey. III: Efferent cortical output and comments on function. *Journal of Comparative Neurology, 212*(1), 38-52.	38	646	17.9
68	Büchel, C., Morris, J., Dolan, R. J., & Friston, K. J. (1998). Brain systems mediating aversive conditioning: an event-related fMRI study. *Neuron, 20*(5), 947-957.	58	643	32.2
69	Lamm, C., Decety, J., & Singer, T. (2011). Meta-analytic evidence for common and distinct neural networks associated with directly experienced pain and empathy for pain. *NeuroImage, 54*(3), 2492-2502.	73	638	91.1
70	Coghill, R. C., Sang, C. N., Maisog, J. M., & Iadarola, M. J. (1999). Pain intensity processing within the human brain: a bilateral, distributed mechanism. *Journal of Neurophysiology, 82*(4), 1934-1943.	59	635	33.4
71	Winston, J. S., Strange, B. A., O'Doherty, J., & Dolan, R. J. (2002). Automatic and intentional brain responses during evaluation of trustworthiness of faces. *Nature Neuroscience, 5*(3), 277-283.	50	628	39.3
72	Paulus, M. P., & Stein, M. B. (2006). An insular view of anxiety. *Biological Psychiatry, 60*(4), 383-387.	33	626	52.2
73	Berendse, H. W., Galis-De Graaf, Y., & Groenewegen, H. J. (1992). Topographical organization and relationship with ventral striatal compartments of prefrontal corticostriatal projections in the rat. *Journal of Comparative Neurology, 316*(3), 314-347.	139	623	24.0
74	Hölzel, B. K., Lazar, S. W., Gard, T., Schuman-Olivier, Z., Vago, D. R., & Ott, U. (2011). How does mindfulness meditation work? Proposing mechanisms of action from a conceptual and neural perspective. *Perspectives on Psychological Science, 6*(6), 537-559.	243	620	88.6
75	Lamm, C., Batson, C. D., & Decety, J. (2007). The neural substrate of human empathy: effects of perspective-taking and cognitive appraisal. *Journal of Cognitive Neuroscience, 19*(1), 42-58.	70	614	55.8
76	Goldman, R. I., Stern, J. M., Engel Jr, J., & Cohen, M. S. (2002). Simultaneous EEG and fMRI of the alpha rhythm. *NeuroReport, 13*(18), 2487-2492.	29	613	38.3
77	Lazar, S. W., Kerr, C. E., Wasserman, R. H., Gray, J. R., Greve, D. N., Treadway, M. T., …& Rauch, S. L. (2005). Meditation experience is associated with increased cortical thickness. *NeuroReport, 16*(17), 1893-1897.	25	612	47.1
78	Myers, K. M., & Davis, M. (2002). Behavioral and neural analysis of extinction. *Neuron, 36*(4), 567-584.	145	609	38.1
79	Zatorre, R. J., Evans, A. C., & Meyer, E. (1994). Neural mechanisms underlying melodic perception and memory for pitch. *Journal of Neuroscience, 14*(4), 1908-1919.	72	606	25.3
80	Downar, J., Crawley, A. P., Mikulis, D. J., & Davis, K. D. (2000). A multimodal cortical network for the detection of changes in the sensory environment. *Nature Neuroscience, 3*(3), 277.	31	600	33.3
81	Fink, G. R., Markowitsch, H. J., Reinkemeier, M., Bruckbauer, T., Kessler, J., & Heiss, W. D. (1996). Cerebral representation of one's own past: neural networks involved in autobiographical memory. *Journal of Neuroscience, 16*(13), 4275-4282.	80	600	27.3
82	Jackson, P. L., Meltzoff, A. N., & Decety, J. (2005). How do we perceive the pain of others? A window into the neural processes involved in empathy. *NeuroImage, 24*(3), 771-779.	79	592	45.5
83	Knutson, B., Westdorp, A., Kaiser, E., & Hommer, D. (2000). FMRI visualization of brain activity during a monetary incentive delay task. *NeuroImage, 12*(1), 20-27.	36	585	32.5
84	Calvert, G. A. (2001). Crossmodal processing in the human brain: insights from functional neuroimaging studies. *Cerebral Cortex, 11*(12), 1110-1123.	95	584	34.4
85	Makris, N., Kennedy, D. N., McInerney, S., Sorensen, A. G., Wang, R., Caviness Jr, V. S., & Pandya, D. N. (2004). Segmentation of subcomponents within the superior longitudinal fascicle in humans: a quantitative, in vivo, DT-MRI study. *Cerebral Cortex, 15*(6), 854-869.	102	582	44.8
86	Price, C. J. (2010). The anatomy of language: a review of 100 fMRI studies published in 2009. *Annals of the New York Academy of Sciences, 1191*(1), 62-88.	135	581	72.6
87	Bush, G., Frazier, J. A., Rauch, S. L., Seidman, L. J., Whalen, P. J., Jenike, M. A., …& Biederman, J. (1999). Anterior cingulate cortex dysfunction in attention-deficit/hyperactivity disorder revealed by fMRI and the Counting Stroop. *Biological Psychiatry, 45*(12), 1542-1552.	86	581	30.6
88	Coghill, R. C., Talbot, J. D., Evans, A. C., Meyer, E., Gjedde, A., Bushnell, M. C., & Duncan, G. H. (1994). Distributed processing of pain and vibration by the human brain. *Journal of Neuroscience, 14*(7), 4095-4108.	121	581	24.2
89	Garavan, H., Pankiewicz, J., Bloom, A., Cho, J. K., Sperry, L., Ross, T. J., …& Stein, E. A. (2000). Cue-induced cocaine craving: neuroanatomical specificity for drug users and drug stimuli. *American Journal of Psychiatry, 157*(11), 1789-1798.	45	576	32.0
90	Naqvi, N. H., Rudrauf, D., Damasio, H., & Bechara, A. (2007). Damage to the insula disrupts addiction to cigarette smoking. *Science, 315*(5811), 531-534.	29	573	52.1
91	Murphy, F. C., Nimmo-Smith, I. A. N., & Lawrence, A. D. (2003). Functional neuroanatomy of emotions: a meta-analysis. *Cognitive, Affective, & Behavioral Neuroscience, 3*(3), 207-233.	229	571	38.1
92	Vertes, R. P. (1991). A PHA-L analysis of ascending projections of the dorsal raphe nucleus in the rat. *Journal of Comparative Neurology, 313*(4), 643-668.	141	567	21.0
93	Phelps, E. A., O'Connor, K. J., Gatenby, J. C., Gore, J. C., Grillon, C., & Davis, M. (2001). Activation of the left amygdala to a cognitive representation of fear. *Nature Neuroscience, 4*(4), 437-441.	28	565	33.2
94	Craig, A. D., Chen, K., Bandy, D., & Reiman, E. M. (2000). Thermosensory activation of insular cortex. *Nature Neuroscience, 3*(2), 184-190.	50	561	31.2
95	Phillips, M. L., Young, A. W., Scott, S., Calder, A. J., Andrew, C., Giampietro, V., …& Gray, J. A. (1998). Neural responses to facial and vocal expressions of fear and disgust. *Proceedings of the Royal Society of London B: Biological Sciences, 265*(1408), 1809-1817.	41	554	27.7
96	Johnstone, T., van Reekum, C. M., Urry, H. L., Kalin, N. H., & Davidson, R. J. (2007). Failure to regulate: counterproductive recruitment of top-down prefrontal-subcortical circuitry in major depression. *Journal of Neuroscience, 27*(33), 8877-8884.	52	553	50.3
97	Kuhnen, C. M., & Knutson, B. (2005). The neural basis of financial risk taking. *Neuron, 47*(5), 763-770.	37	550	42.3
98	Reiman, E. M., Lane, R. D., Ahern, G. L., Schwartz, G. E., Davidson, R. J., Friston, K. J., …& Chen, K. (1997). Neuroanatomical correlates of externally and internally generated human emotion. *American Journal of Psychiatry, 154*(7), 918-925.	45	550	26.2
99	Weiller, C., Ramsay, S. C., Wise, R. J., Friston, K. J., & Frackowiak, R. S. (1993). Individual patterns of functional reorganization in the human cerebral cortex after capsular infarction. *Annals of Neurology, 33*(2), 181-189.	40	545	21.8
100	Quirk, G. J., Likhtik, E., Pelletier, J. G., & Paré, D. (2003). Stimulation of medial prefrontal cortex decreases the responsiveness of central amygdala output neurons. *Journal of Neuroscience, 23*(25), 8800-8807.	62	544	36.3

For the 67 original articles, 57 used human subjects whereas 10 used animal models. MRI was the commonest modality (*n* = 37), followed by PET (*n* = 16). Nine articles investigated by histology, two by multiple modalities and three by other modality.

Citation count was significantly correlated with normalized citation count (*r* = 0.840, *p* < 0.001), reference count (*r* = 0.289, *p* = 0.004), usage count (*r* = 0.702, *p* < 0.001) but not years since publication (*r* = −0.161, *p* = 0.109), number of authors (*r* = −0.071, *p* = 0.482), and page count (*r* = 0.172, *p* = 0.087). Averaged citation count of open access publications (293.2, *SD* = 435.1) did not differ from that of non-open access counterparts (970.4, *SD* = 542.0) (*p* = 0.470). Meta-analyses/reviews generally had more citations than experiments/lab studies (1,171.5 vs. 831.0, *p* = 0.004). Articles reporting findings from normal subjects had more citations than those reporting from patients with diseases/disorders (991.1 vs. 752.5, *p* = 0.003). Articles using human model had more citations than those using animal models (857vs. 678.8, *p* = 0.009). Meanwhile, MRI (906.1, *SD* = 539.6) and non-MRI (738.5, *SD* = 213.3) articles did not have significant difference in their average citation counts (*p* = 0.090).

### Major contributors

The citation classics were contributed by 361 authors working in 130 affiliations in 13 countries. They were published in 38 journals. The five major contributing affiliations were Stanford University (*n* = 8), Harvard University (*n* = 8), University College London (*n* = 6), Massachusetts General Hospital, National Institute of Mental Health, University of Iowa and Washington University (each *n* = 5). The top five major contributing countries were USA (*n* = 70), UK (*n* = 27), Canada (*n* = 11), Germany (*n* = 6), and Italy (*n* = 5). The top five major contributing journals were Journal of Neuroscience (*n* = 8), Journal of Comparative Neurology (*n* = 7), Biological Psychiatry, NeuroImage, and Neuron (each *n* = 6). The top five major WoS categories to which the citation classics were assigned were neurosciences (*n* = 79), multidisciplinary sciences (*n* = 14), clinical neurology (*n* = 13), psychiatry (*n* = 13), and neuroimaging, radiology/ nuclear medicine/ medical imaging and zoology (each *n* = 7). Note that each publication could be assigned to multiple WoS categories.

### Bubble map visualizing terms in titles and abstracts

**Figure 1:**
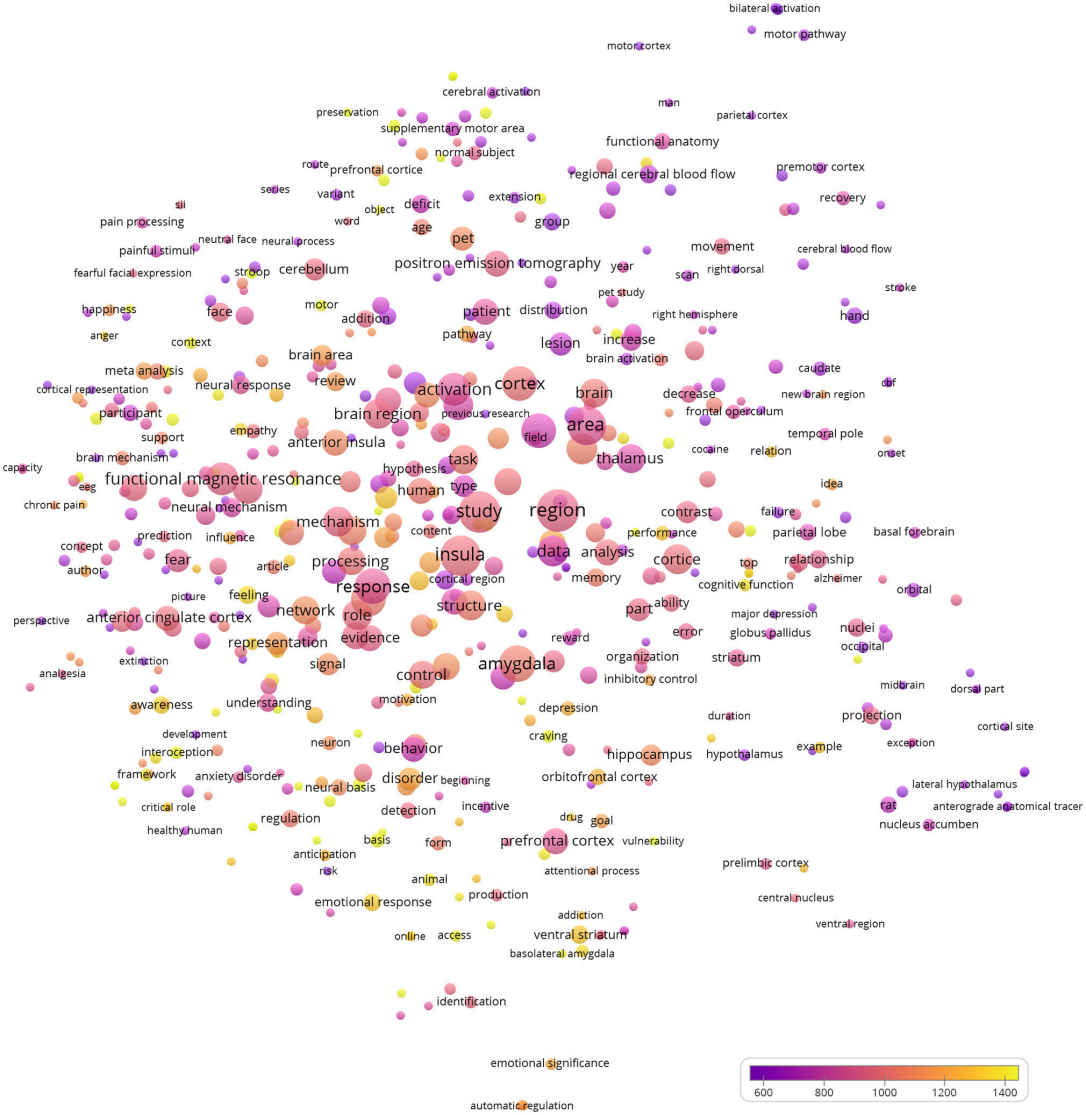
Bubble map visualizing the words/phrases used in the titles and abstracts of the 100 most cited publications concerning the insular cortex of the brain. All words/phrases that appeared in two or more of the 100 publications were included. Binary counting was used, implying that multiple appearances in a single publication counted as one. Default visualization parameters of the software VOSviewer were chosen. Two bubbles were in closer proximity if they co-appeared in a larger number of publications. The number of publications in which the word/phrase appeared determined the bubble size. The bubble color was determined by the averaged citations per publication containing the word/phrase.

Figure 1 shows the bubble map that visualizes the words/phrases used in the titles and abstracts of the 100 publications. Various aspects of the publications might be observed. First, functional magnetic resonance imaging (appearances = 24, average citations = 937.2) and positron emission tomography (appearances = 15, average citations = 944.1) were the two main imaging modalities. When the underlying data of the bubble map was examined, computed tomography, the popular modality used in medical field, was absent. This has implied that the most cited publications on the insula have been focusing on functional neuroimaging heavily.

Second, the bubble representing insula was close to bubbles of words related to functions, such as mechanism (appearances = 20, average citations = 951.9), processing (appearances = 18, average citations = 997.2), response (appearances = 29, average citations = 893.7), role (appearances = 20, average citations = 970.1), activation (appearances = 25, average citations = 891.5), and control (appearances = 18, average citations = 992.1). Certainly, the structure (appearances = 20, average citations = 1031.3) of the insula was also one of the main involved topics.

Third, brain regions that were often mentioned together with the insula (10 times or more) included the amygdala (appearances = 31, average citations = 1027.7), thalamus (appearances = 19, average citations = 833.4), anterior cingulate (appearances = 17, average citations = 993.1) and prefrontal cortex (appearances = 15, average citations = 911.0), cerebellum (appearances = 11, average citations = 958.2), and hippocampus (appearances = 10, average citations = 1087.2).

## Discussion

The current study has revealed that the citation classics concerning the insula were published across a long period of time spanning nearly four decades. Most of them were original articles and contributed by authors working in affiliations based in USA. Moreover, most of them were classified by WoS as dealing with neurosciences, and some of them as clinical neurology, psychiatry and neuroimaging. However, it should be noticed that seven of them were classified as dealing with zoology. This was due to the fact that Journal of Comparative Neurology encourages submissions that compare brain functions among species, also known as systems neuroscience, that can be considered as relevant to zoology. The large share of citation classics contributed by Journal of Comparative Neurology reminds us the importance and usefulness of investigations using different species as models, such as monkeys and rats.

Though all of the publications were dealing with the insula, it could be observed that brain regions often mentioned together with the insula included the amygdala, thalamus, anterior cingulate and prefrontal cortex, cerebellum, and hippocampus. For instance, the top-ranked most cited insula publication investigated gray matter volume loss in aging adults, and reported that the insula and cingulate were affected but not the amygdala and the hippocampus (Good et al., 2001). Meanwhile, the 2nd-ranked most cited insula publication reported that the anterior cingulate and orbital frontoinsular cortices were involved in a salience network that linked to limbic and subcortical regions, and an executive network that communicated with prefrontal and parietal cortices (Seeley et al., 2007). A previous bibliometric report has also pointed out that neuroimaging studies on food stimulations generally had more citations if the insula, orbitofrontal cortex and amygdala were involved (Yeung, 2018). These findings have reflected that notion that the insula is an integration hub that connects multiple brain regions. Besides, Table 1 has shown that the most top-ranked publications were concerning emotion, salience and pain. One possible reason for articles investigating normal brain functions/mechanisms from healthy subjects having more citations than those investigating patients was that findings from normal subjects might be more generalizable and applicable to a larger population.

The positive correlation between citation count and reference count is consistent to many previous studies (Peters and Van Raan, 1994; Lokker et al., 2008; Farshad et al., 2013; Hanssen and Jørgensen, 2014; Fox et al., 2016). However, the Pearson correlation coefficient was very small (0.289). It implied that if a larger dataset or a different dataset was examined, it might become insignificant. The non-correlation between citation count and year since publication is also consistent with previous findings (Hanel and Haase, 2017). The current sample was small and not random, but the results of such exploratory analysis seemed to suggest that the selected insula literature did not deviate much from other representative body of literature with regards to influencing factors of citation count. The non-correlation between citation count and author count, page count or open access status, however, are inconclusive because previous studies have reported mixed results (see Table 1 of Hanel and Haase, 2017).

The most important limitation of the current study was that bibliometric analysis cannot assess the validity of or the level of scientific evidence reported by the analyzed publications. A highly cited article may not necessarily have high scientific quality. Moreover, the citation count received by a publication might depend on other factors that might not be fully considered in the current study. To start with, an article may be cited more if it was published in a more visible journal (Brink, 2013); For example, articles published in Chinese scientific journals tended to have few citations and thus leading to low journal impact factors (Ren and Rousseau, 2002). Meanwhile, the study model might also be an influencing factor. It was reported that basic research generally received more citations than clinical studies (Van Eck et al., 2013); and methods papers were often cited as a common practice so they accumulated many citations (Van Noorden et al., 2014). In the current study, human studies on average had 179 more citations than animal studies. The imaging modality may also be a factor, as a previous bibliometric study has reported that most of the highly cited neuroimaging papers used MRI compared to PET, computed tomography and ultrasonography (Kim et al., 2016). The current study also found that MRI was the commonest modality, but its usage did not significant influence citation count. Finally, there could be authors who self-cited a lot, which might then lead to an increased visibility of the articles and hence more citations from others (Fowler and Aksnes, 2007). Readers should be clearly aware of these confounding factors when they interpret the results from the current manuscript. Also, it is intuitive to reason that the citation counts from older publications should usually be larger than more recent publications, thus making the ranking and analyses biased. Nonetheless, the current results have demonstrated a strong positive correlation between total and normalized citation counts. Maybe it was partly due to the fact that the latest publication among the most cited 100 publications has already been published for 7 years. Such a long period of time might have already allowed publications to cumulate a large number of citations, as it was reported that the number of annual citations usually tended to reach a maximum of plateau 2–3 years after publication (Hansen and Henrikson, 1997).

To conclude, the current study has identified and analyzed the 100 most cited publications concerning the insula of the brain. These publications have shown that the insula is a multifunctional brain region. The top ranked papers on the list tended to focus on emotion, salience and pain. Brain regions often mentioned together with the insula included the amygdala, thalamus, anterior cingulate and prefrontal cortex, cerebellum, and hippocampus. Citation count of these 100 highly cited publications correlated with their normalized citation count, number of references and usage count in WoS, but not years since publication, number of authors, page count and status of open access.

## Author contributions

The author confirms being the sole contributor of this work and approved it for publication.

### Conflict of interest statement

The author declares that the research was conducted in the absence of any commercial or financial relationships that could be construed as a potential conflict of interest.
